# Sticking to it: phytopathogen effector molecules may converge on evolutionarily conserved host targets in green plants

**DOI:** 10.1016/j.pbi.2018.04.019

**Published:** 2018-08

**Authors:** Philip Carella, Edouard Evangelisti, Sebastian Schornack

**Affiliations:** University of Cambridge, Sainsbury Laboratory, Cambridge, United Kingdom

## Abstract

• Phytopathogen effectors converge on similar sets of host proteins in angiosperms.
• Effectors may target host proteins and processes present across the green plant lineage.
• Bryophyte model plants are promising systems to investigate effector–target relationships.

Plant-associated microbes secrete effector proteins that subvert host cellular machinery to facilitate the colonization of plant tissues and cells. Accumulating data suggests that independently evolved effectors from bacterial, fungal, and oomycete pathogens may converge on a similar set of host proteins in certain angiosperm models, however, whether this concept is relevant throughout the green plant lineage is unknown. Here, we explore the idea that pathogen effector molecules target host proteins present across evolutionarily distant land plant lineages to promote disease. We discuss that host proteins targeted by phytopathogens or integrated into angiosperm immune receptors are likely found across green plant genomes, from early diverging non-vascular lineages (bryophytes) to flowering plants (angiosperms). This would suggest that independently evolved pathogens might manipulate their hosts by targeting `vulnerability’ hubs that are present across land plants. Future work focusing on accessible early divergent land plant model systems may therefore provide an insightful evolutionary backdrop for effector–target research.

## Graphical abstract

**Figure:**
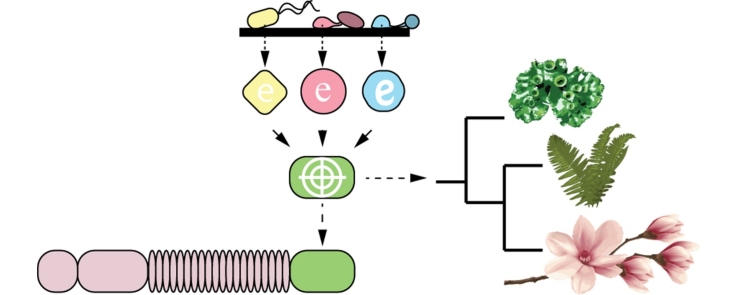

**Current Opinion in Plant Biology** 2018, **44**:175–180

This review comes from a themed issue on **Biotic interactions**

Edited by **Sebastian Schornack** and **Caroline Gutjahr**

For a complete overview see the Issue and the Editorial

Available online 4th July 2018

**https://doi.org/10.1016/j.pbi.2018.04.019**

1369-5266/© 2018 The Authors. Published by Elsevier Ltd. This is an open access article under the CC BY license (http://creativecommons.org/licenses/by/4.0/).

## Introduction

**Figure 1:**
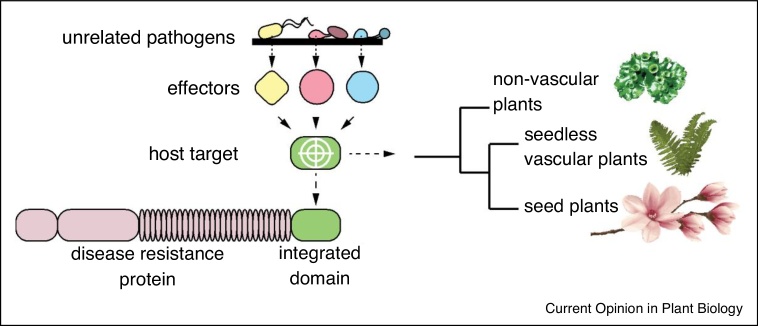
A conceptualized diagram highlighting the idea that host proteins targeted by unrelated pathogens and the integrated domains of disease resistance (R) proteins are present across the green plant lineage.

The green plant lineage (Embryophytes) evolved from freshwater charophyte green algae over 450 million years ago and has since dominated terrestrial environments [1,2^•^]. The evolutionary history and diversification of land plant lineages is extensively reviewed elsewhere [3, 4, 5], but generally follows that non-vascular gametophyte-dominant bryophytes (liverworts, hornworts, mosses) were the earliest diverging lineage whose ancestor gave rise to sporophyte-dominant vascular plants (tracheophytes) that include lycophytes (clubmosses), ferns, and seed plants, which themselves diverged to gymnosperms (non-flowering) and angiosperms (flowering). Throughout their evolutionary history, plants have been exposed to a diverse range of microbial life forms that had the potential to impact their fitness. To protect themselves from detrimental microbes, land plants utilize a tiered immune system that includes the detection of common microbial motifs (MAMPs, microbe-associated molecular patterns), such as bacterial flagellin or fungal chitin, via membrane-localized PRRs (pattern recognition receptors) as an early line of defence [6,7^•^]. The recognition of MAMPs by PRRs initiates an intracellular MAP kinase signalling cascade that activates MTI (MAMP-triggered immunity), leading to several well-described molecular and physiological adjustments that limit pathogen ingress [6,8,9]. Conversely, microbes evolved effector proteins that suppress MTI and other host cellular activities to render hosts susceptible and promote disease [10,11,12^•^]. To date, pathogen effector research is largely performed in angiosperms, which represent an evolutionarily young (albeit diverse) land plant lineage. Below, we introduce key concepts of effector biology obtained from angiosperm-based pathosystems and project this knowledge onto earlier diverging land plant lineages to explore the idea that effectors target evolutionarily conserved plant proteins and processes (Figure 1).

## Back to basics: key concepts in effector biology

Pathogen effector molecules are translocated into host tissues and cells, where they target important macromolecules (proteins, cell wall components, nucleic acids, and so on) involved in normal cellular functions and/or immunity [10,11,12^•^]. Effectors are generally catalogued into two distinct groups; those acting in the extracellular spaces of host tissues (apoplastic) or those acting within host cells (cytoplasmic). Apoplastic effectors are secreted via general eukaryotic secretion systems in oomycetes/fungi [13,14^•^] or via the type II secretion system (T2SS) of bacterial pathogens [15]. These molecules are typically involved in the enzymatic degradation of plant cell walls, immune evasion, or the suppression of host proteolytic activity [11,16]. Bacterial pathogens such as *Pseudomonas syringae* and *Xanthomonas* spp. inject cytoplasmic effectors directly into plant cells using a specialized type III secretion system (T3SS) and are hence termed ‘type III effectors’ [12^•^,^15^]. In comparison, our understanding of how the cytoplasmic effectors of eukaryotic filamentous microbes are delivered into plant cells remains unclear, however it is generally believed that certain effector families (i.e. RXLR and CRN/crinkler) enter and act within host cells [10,11,17]. These molecules are likely delivered through specialized hyphal structures that invaginate plant cells (haustoria), or perhaps are endocytosed from the apoplast [18,19]. Cytoplasmic effectors have been extensively studied in angiosperms, revealing a suite of virulence strategies wherein effectors access various cellular compartments (nucleus, chloroplast, cytoplasm, and so on) to disrupt the activity of host proteins involved in transcriptional regulation, secretion, metabolism, programmed cell death, and hormone signalling [10,11,12^•^,^20^].

## Unrelated effector molecules may converge on similar networks of host proteins

Investigating phytopathogen effector function is typically implemented in a formulaic manner, where individual effectors displaying activity in plant cells (i.e. disease promotion/immune suppression) are used as baits to identify host targets in yeast 2-hybrid or immunoprecipitation-mass spectrometry (IP-MS) screens (discussed in [17]). This is then followed by more detailed analyses to validate candidate interactors and determine how the effector acts to facilitate disease progression. While highly effective, this approach provides only a snapshot of a given host–microbe interaction, as phytopathogens can deliver anywhere from 30 to 80 type III effectors (bacterial pathogens) or in some cases over 200 cytoplasmic effectors (fungi/oomycetes). It was therefore crucial that a more exhaustive screen for effector targets be conducted. Seminal studies carried out by Mukhtar *et al*. [21^••^] and Wessling *et al*. [22^••^] describe high-throughput yeast 2-hybrid screens and network analysis of candidate oomycete (*Hyaloperonospora arabidopsidis*), fungal (*Golovinomyces orontii*), and bacterial (*P. syringae*) effectors in the model angiosperm *Arabidopsis thaliana.* Together, these works revealed a core hub of plant proteins that are likely targeted by unrelated phytopathogens, which suggests that effectors may converge onto these hubs to promote microbial fitness *in planta* (Figure 1). While experimental evidence confirming interactions/modulation of homologous plant targets with individual phytopathogen effectors is lacking on a large scale *in vivo*, limited evidence supports the possibility of such a scenario. For example, the immune-regulator SGT1 associates with the *Ustilago maydis* (fungus) effector SEE1 in maize and with the *Xanthomonas campestris* (bacterial) AvrBsT effector in pepper [23, 24, 25]. Moreover, the TCP14 transcription factor, which was found to interact with multiple effectors in the yeast 2-hybrid screening described in Ref. [22^••^], was also shown to interact with the *Phytophthora capsici* (oomycete) CRN12-997 effector in tomato and the *P. syringae* (bacterial) effector HopBB1 in Arabidopsis [26,27]. Whether such convergent targeting holds true over a diverse range of plant–pathogen interactions remains to be determined. If so, it would imply that effectors manipulate conserved host proteins to promote disease progression in land plants (Box 1).

Box 1 **Are effectors deployed during interactions with early diverging land plants?**

Our current understanding of plant–microbe interactions is heavily skewed toward angiosperm models, with comparatively less known about how microbes interact with early diverging land plant lineages. This is especially true for plant–pathogen interactions, which are under-represented compared to interactions between early diverging land plants and symbiotic microbes [44,59]. Evidence for the direct action of effector molecules in early diverging lineages is lacking, yet several lines of evidence suggest that phytopathogens use effectors to manipulate these plants. For example, several plant lineages (mosses, liverworts, ferns) are amenable to *Agrobacterium*-mediated transformation, which requires the successful delivery of transfer (T)-DNA by effector molecules that travel together through the bacterial type IV secretion system [15,60, 61, 62]. Moreover, putative apoplastic effectors and toxins from the necrotrophic bacterial pathogen *Pectobacterium caratovorum* were shown to induce cell death in the moss *P. patens*. To our knowledge, the action of cytoplasmic effectors in moss has not yet been described, however, hemi-biotrophic pathogens that deploy effectors in angiosperms (*Phytophthora, Colletotrichum*) successfully colonize moss [63,64^•^] and likely do so using effectors. Moreover, we recently reported that the hemi-biotrophic oomycete pathogen *P. palmivora* establishes digit and branched intracellular haustoria-like structures in *M. polymorpha* liverwort cells, which was associated with the upregulation of apoplastic and cytoplasmic (predominantly RXLR) effector molecules [65^••^]. Together, these studies hint at the importance of phytopathogen effectors in manipulating diverse land plant lineages. Future efforts to understand the extent to which effectors of broad-host range pathogens modulate liverwort, angiosperm, and perhaps even lycophyte/fern susceptibility will be of particular importance in exploring the conservation/convergence of effector–host relationships.

Alt-text: Box 1

## Are angiosperm proteins and processes commonly targeted by phytopathogen effectors also present in evolutionarily distant green plants?

Our current understanding of how phytopathogen effectors manipulate host plants is primarily focused on pathogenic interactions with angiosperms. The lack of experimental knowledge on effector–target relationships in non-flowering plants therefore limits comparative analyses with angiosperms, however as a first step we can assess whether known proteins and processes targeted by effectors are present across land plants. Recent phylogenetic analyses demonstrate that the general immune regulators SGT1, RAR1, and HSP90, which form a complex to regulate immune protein stability in angiosperms [28], are conserved across diverse land plant lineages that include early divergent bryophytes (*Marchantia polymorpha, Physcomitrella patens*), a vascular non-flowering lycophyte (*Selaginella moellendorffii*), a gymnosperm seed plant (*Picea abies*), and an early divergent angiosperm (*Amborella trichopoda*) [29^••^]. Given that several unrelated phytopathogen effectors (*U. maydis* SEE1, *X. campestris* AvrBsT, *P. syringae* AvrB, *Phytophthora sojae* CRN108) have been shown to modulate this complex [23, 24, 25,30], it is plausible that adapted or broad-host pathogens may employ effectors that target the conserved SGT1–RAR1–HSP90 complex in non-flowering plants. This idea can also be extended to the exocyst complex, MAP kinase cascades, TCP transcription factors, and the defense hormone salicylic acid (SA). The exocyst complex, and vesicular trafficking in general, is targeted by several unrelated phytopathogen effectors in angiosperms (*Phytophthora infestans* AVR1, *Magnaportha oryzae* AVR-Pii, *P. syringae* HopM1) [31, 32, 33] and is broadly conserved in the green plant lineage [34]. MAP kinases regulating innate plant immunity are similarly conserved across land plants [7^•^,29^••^] and appear to be recurring targets of effectors during pathogen–angiosperm interactions (*P. syringae* AvrB, HopAI1, HopF2, HopZ3; *P. infestans* PexRD2, Pi17316) [35, 36, 37, 38, 39, 40, 41]. The plant-specific TCP transcription factor family is also present across the green plant lineage [29^••^] and, as described above, is likely targeted by unrelated pathogen effectors. Lastly, the defense hormone salicylic acid (SA), which is commonly manipulated by various effectors in angiosperms (via enzymatic degradation or inhibition of signalling pathways; reviewed in [11,42]), has been detected in early divergent land plants that encode homologs of core SA synthesis and signalling genes [29^••^,^43^, ^44^, ^45^]. Collectively, the accumulated evidence suggests that homologs of core immune, cell biology, and hormonal pathways are at the very least present across divergent land plant lineages, which is a prerequisite for the potential convergence of effectors onto these hubs/processes. Given their importance for plant–pathogen interactions in a broad range of angiosperm species, we expect that these pathways represent likely targets for pathogen effectors across green plant lineages.

## Why target conserved proteins and processes?

Conceptually, the targeting of conserved plant proteins and processes by phytopathogen effectors would provide flexibility in the expansion of a pathogen’s host range and/or could facilitate evolutionary transitions or `shifts’ to new hosts. In such a scenario, a pathogen would possess a basic set of effectors required to colonize a plant and additional genomic innovations could then ensure the successful colonisation of a specific host species. Surprisingly, virulent isolates of the phytopathogen *P. syringae* share only a few type III effectors between the majority of host-specific strains [46^•^], which appear to be involved in the promotion of an aqueous extracellular environment and the suppression of early immune responses [47]. However, host-shifting could still occur if non-conserved type III effectors target conserved plant proteins/processes. Indeed, some *P. syringae* effectors (AvrB, AvrPto, HopAI1) have been shown to target conserved host proteins present in both Arabidopsis and tomato (reviewed in [12^•^]). By contrast, filamentous oomycete pathogens belonging to the genus *Phytophthora* can sometimes display a strikingly broad host range (i.e. *P. palmivora*), with the ability to colonize and complete its asexual life cycle in several monocot, dicot, and even bryophyte hosts (discussed in [44,48]). Whether individual effectors evolved to target broadly conserved plant proteins remains to be determined, but this would begin to explain the success of broad host-range *Phytophthora* species.

Exactly why diverse land plant lineages have conserved proteins commonly targeted by phytopathogen effectors likely has to do with the overall importance of these proteins for normal growth and development, or for immune responses to a greater number of microbial threats. This may be true for certain effector targeted host proteins that are heavily interconnected to various processes/proteins (so-called ‘immune hubs’ [21^••^,22^••^]), where the activity of effectors (either alone or in concert) are thought to modulate the functionality of the entire hub to promote disease. Such hub proteins appear to be involved in essential processes and have strong structural and/or sequence constraints that cannot be modified or removed without an overall impairment to plant fitness. As described above, certain effector-targeted hubs/complexes can also be found in evolutionarily distant green plant lineages, which is not surprising given that these proteins play important roles in plant metabolism, cell biology, and development. How this impacts host-range and specificity remains to be determined, but an effector repertoire capable of targeting broadly conserved proteins/processes is likely advantageous since the corresponding host targets would be present across a wide range of potential hosts.

### Mastering your domains: new resistance strategies shed light on older targets

The inability to remove or modify hub proteins targeted by phytopathogen effectors necessitated the development of alternative means to mitigate effector activities. Many plants escape effector-mediated susceptibility using disease resistance (R) proteins that are able to detect pathogen effector molecules (or their activity on hubs) through a multitude of molecular strategies (discussed in [49]). This includes molecular decoys of effector-targeted protein domains that either associate with plant R protein complexes or are directly integrated into R proteins themselves (Figure 1). Using this strategy, plants effectively set molecular traps for cognate effectors, such that any activity on the decoy/integrated domain of an R protein activates the robust effector-triggered immunity (ETI) response [50,51^•^]. Previous studies have reasoned that the diverse array of integrated domains in otherwise structurally similar R proteins (also called NLRs: nucleotide-binding domain, leucine rich repeats) likely represent former stand-alone targets of phytopathogen effectors [51^•^,^52^]. Intriguingly, candidate integrated domain-containing R proteins are present in early divergent land plants [29^••^,51^•^], which suggests that this strategy is broadly employed across the green plant lineage, although only a few early divergent plant genomes have been sequenced thus far. This brings forth interesting questions as to how and when effector targets were integrated into R proteins (Figure 1). The number of NLRs encoded by early divergent land plants appears to be limited but includes candidates that contain protein kinases or TPR (tetracotripeptide repeat) domains in *P. patens* (moss), CCT (**C**ONSTANS, **C**O-like, **T**OC1) or TIFY (TIF[*F*/*Y*]XG) domains in *S. moellendorffii* (lycophyte), and α/β-hydrolases in *M. polymorpha* (liverwort) [51^•^,^53^,^54^]. These domains may hint to relevant effector-targeted immunity proteins in these lineages (discussed in [44]). Integrated domains of angiosperm R proteins may also represent putative effector targets already present in the common ancestor of different plant lineages, which further underscores the need to extend molecular plant–microbe interaction research to evolutionarily distant land plants [55]. Taken together, these observations support the idea that host proteins and processes targeted by phytopathogen effectors may be conserved across the green plant lineage and suggests that broad evolutionary insights can be gained by studying pathogenic interactions in divergent land plant lineages.

### What’s on the horizon: exploring effector–target conservation using evolutionarily distant land plant models

Detailed evolutionary analyses are required to address whether host proteins targeted by phytopathogen effectors are indeed conserved across land plants, which likely requires a greater understanding of plant–microbe interactions across a more diverse range of hosts and microbes. The conservation of effector targets across green plants would raise important evolutionary questions on the role of effectors in plant–microbe interactions. For example, to what extent do pathogens utilize effectors that converge on broadly conserved plant proteins/processes, such as those involved in secretion, and is this indicative of a crucial vulnerability in plants that was exploited early during the evolutionary history of pathogenic microbes? Do individual effectors interact with highly conserved host proteins in divergent land plant species and, if so, do they maintain their activity? How frequently do effectors target conserved proteins, and is this more prevalent in broad-host range pathogens, or perhaps microbes with smaller effector repertoires? While it is clear that additional experimentation is required to address these questions, an immediate practical benefit of this paradigm is that it opens the door to more amenable genetic systems. Bryophytes like the moss *P. patens* and the liverwort *M. polymorpha* are ideal candidates for this given their haploid-dominant lifestyle, reduced genetic redundancy, availability of molecular genetic tools (targeted mutagenesis; efficient transformation protocols; binary vector sets), and their ability to interact with filamentous pathogens [44,45,55, 56, 57]. Indeed, effector research has even been successful in non-plant models such as yeast (*Saccharomyces cerevisiae*), where the conserved action of bacterial type III effectors on MAP kinase signalling and other cellular processes has been demonstrated under normal or stress conditions [58]. We therefore expect that the use of bryophytes in this context will aid future endeavors to identify and characterize phytopathogen effector targets that will likely be relevant to angiosperm crop–microbe interactions. This may ultimately lead to the identification of broadly conserved vulnerability hubs that could be safeguarded to protect important crop plants.

## References and recommended reading

Papers of particular interest, published within the period of review, have been highlighted as:

• of special interest

•• of outstanding interest
